# Intimate partner violence across pregnancy and the postpartum and the relationship to depression and perinatal wellbeing: findings from a pregnancy cohort study

**DOI:** 10.1007/s00737-024-01455-z

**Published:** 2024-03-09

**Authors:** Megan Galbally, Stuart Watson, Kelli MacMillan, Katherine Sevar, Louise M Howard

**Affiliations:** 1 School of Clinical Sciences, Monash University, Clayton, Australia; 2 Health Futures Institute, Murdoch University, Perth, Australia; 3 Health Services and Population Research Department, Institute of Psychiatry, Psychology and Neurosciences, King’s College London, London, UK; 4 Monash Health, Clayton, VIC Australia

**Keywords:** Depression, Pregnancy, Intimate partner violence, Childhood trauma

## Abstract

**Purpose:**

To compare the prevalence of emotional and physical intimate partner violence (IPV) across pregnancy and the first year postpartum in those with and without clinical depression and assess the association between maternal childhood trauma, current stressful life events and depression and IPV over the perinatal period.

**Methods:**

Data were obtained from 505 pregnant women from the Mercy Pregnancy and Emotional Wellbeing Study (MPEWS), a cohort study with data collected across pregnancy until 12 months postpartum. Maternal antenatal depression was measured using the Structured Clinical Interview for DSM-IV (SCID-IV) with repeat measurement of perinatal depressive symptoms using the Edinburgh Postnatal Depression Scale (EPDS). Trauma was measured using the Childhood Trauma Questionnaire, and experiences of physical and emotional intimate partner violence using items in the Stressful Life Events Scale.

**Results:**

Women experiencing IPV across the perinatal period were significantly more likely to score over 13 on the EPDS (*p* < .001) at each timepoint in pregnancy and the postpartum and physical IPV was associated with clinical depression. Further, a history of childhood trauma and current additional stressful life events were significantly associated with reporting current IPV in the perinatal period.

**Conclusions:**

This study confirmed the risk factors of childhood trauma and current stressful life events for reporting experiences of IPV in the perinatal period. Furthermore, women experiencing IPV reported higher depressive symptoms, providing evidence supporting the value of assessing those women who screen higher on the EPDS for IPV. Together these findings also support trauma informed care across pregnancy and the postpartum.

**Supplementary Information:**

The online version contains supplementary material available at 10.1007/s00737-024-01455-z.

## Background

There is increasing evidence to suggest that across child bearing years, women with mental health disorders may disproportionately experience intimate partner violence (IPV), with the experiences of IPV potentially associated with impacts on their mental health and wellbeing (Oram et al. 2022; Suparare et al. 2020; Trevillion et al. 2012). Typically IPV encompasses any behaviours, including physical, sexual, and emotional violence, as well as controlling and coercive behaviours perpetrated against a person by their current or former intimate partner, and in family violence these may also include these behaviours from wider family members (Garcia-Moreno et al. 2006; Oram et al. 2022). Within research, data collected may focus on all forms of IPV or may be limited to specific behaviours and experiences of IPV(Paulson 2022). IPV has been associated with poorer perinatal mental health, that is in pregnancy and across the first year postpartum, and this has included depressive and anxiety symptoms and posttraumatic stress disorder, although very few studies have utilised a clinical diagnostic measure to examine specific mental disorders (Halim et al., 2018; Howard et al. 2013; Paulson 2022). Indeed, the recent Lancet Commission on IPV highlighted the paucity of research undertaken with clinical diagnostic measures of mental disorders and that also have collected data on IPV (Oram et al. 2022). Likewise, a systematic review specifically of IPV and perinatal depression examining longitudinal studies, also identified a gap in studies that had utilised a diagnostic measure and highlighted this as a gap within current research (Paulson 2022) Furthermore, another gap is research that can elucidate the relationship between co-occurring risk factors for poorer perinatal outcomes associated with social adversity by including measures of maternal childhood trauma, IPV, current stressfull life events, as well as perinatal depression (Oram et al. 2022).

The importance of understanding the relationship between IPV and perinatal depression lies in the potential additive impact of IPV on increasing vulnerability to develop perinatal depression (Oram et al. 2022). While longitudinal studies examining IPV and perinatal depression were identified in a recent review, very few utilised repeat measurement of current IPV and repeat measurement of depressive symptoms across pregnancy and the postpartum to be able to examine the temporal relationship between these two factors (Paulson 2022) The perinatal period is a time of greater opportunity for potential health screening for both depression as well as IPV and research to understand the relationship between these will inform the development of future screening practices. Screening in the perinatal period is feasible, as it is facilitated by the increased contact with health professionals including regular antenatal and postnatal checks, and within many maternity services there is now routine screening for perinatal depression and/or IPV (Duchesne et al. 2021; Learman 2018). However, screening for IPV and depression is frequently undertaken separately resulting in siloed pathways for care and support (Boyle et al. 2019; Buist et al. 2007; Howard et al. 2014).

While the ecological model of understanding violence against women, including IPV, suggests societal, community, relationship and individual factors need to be considered, there is research to suggest an important vulnerability for IPV is exposure to childhood maltreatment and this relationship has been found to be stronger in women (Heise 1998; Renner and Slack 2006; Shields et al. 2020) In the perinatal period, a history of childhood maltreatment is associated with both vulnerability to perinatal depression, as well as separately having been associated with current experiences of IPV, suggesting the importance of research examining these three factors (Anderson et al., 2016; Galbally et al., 2019). Experiences of trauma, including current trauma as well as a history of maternal childhood maltreatment, may also impact the experience of health care in pregnancy, such as vaginal and other intimate examinations by clinical staff, the experience of vaginal birth and the presence of staff who the woman is unfamiliar with during birth (Do et al. 2022; Montgomery et al. 2015; Sperlich et al. 2017). While much has been described about the importance of trauma informed care for women in pregnancy and the postpartum, embedding this in clinical practice has been slower (Drexler et al. 2022; Racine et al. 2021; Sperlich et al. 2017). Yet, through an increased understanding of interrelated risk factors of social adversity, including childhood maltreatment, current stressful events, IPV, and depression, an important opportunity may be provided for clinicians across mental health and maternity care to improve care and outcomes for women and their children. This includes through the development of an individualised approach that considers a woman’s care and support needs across these multiple factors, and then adapts the well-articulated and adapted trauma informed principles for this population (Drexler et al. 2022).

The overall study aim is to address some of the gaps in research including the use of diagnostic measure of depression within the design and the use of repeat measures of current depressive symptoms together with repeat measurement of current IPV to examine the temporal relationship over the perinatal period (Oram et al. 2022; Paulson 2022). The first aim of this study seeks to compare the prevalence of IPV for women during the perinatal period in those with a current clinical depressive disorder and those without. Specifically, this aim is to compare between those with and without a current depressive disorder, the prevalence of both current emotional and physical IPV during early and late pregnancy, and at six and 12 months in the first year postpartum. The second aim is to assess the association between maternal childhood trauma, current stressful life events and current depression and current IPV over the perinatal period, and then to model change in the prevalence of IPV over the course of the perinatal period assessing if change is associated with maternal depressive symptoms, measured using the Edinburgh Postnatal Depression Scale (EPDS).

## Methods

### Sample

This sample reported in this study are drawn from the Mercy Pregnancy and Emotional Wellbeing Study (MPEWS), which is a selected pregnancy cohort based in Victoria and Western Australia, Australia where both women with clinical depression as well as women without depression or taking antidepressant medication were recruited. A study protocol can be found in Galbally et al. (2017). The total cohort is 887 women; however, for this study women from the cohort were only included in this sample if they had completed the Childhood Trauma Questionnaire (CTQ) (*N* = 505). There were no significant differences in maternal age, parity, ethnicity, relationship status, education, and major depression at recruitment between women who did and women who did not complete the CTQ. The data used in this paper were collected at four timepoints via both paper-based and online survey (REDCap) methods during the perinatal period: early pregnancy up to 20 weeks, third trimester, and six and 12 months postpartum. Ethics approval for the study was provided by The Mercy Health Human Research Ethics Committee (permit #R08/22) and the South Metropolitan Health Service (permit #2016/192). Eligible women provided their written informed consent prior to participation.

### Measures

#### Intimate partner violence and other stressful life events

Women completed a revised 24-item version of the Stressful Life Events Questionnaire (SLE; (Goodman et al. 1998) validated for pregnancy in Australia (Brown et al. 2011) to assess the incidence of both common and pregnancy-specific life stressors. Examples include major illness or injury, relationship change, employment change and unemployment, and financial strain. The SLE was completed in early pregnancy (capturing events up to one year prior to conception), third trimester (capturing new events since those reported in early pregnancy), and six (new events since third trimester) and 12 months postpartum (new events since six months postpartum). To assess the experience of Intimate Partner Violence (IPV), we used participant responses (*No*/*Yes*) to two specific events in the SLEQ at each timepoint: specifically, these were emotional IPV, “You were humiliated or emotionally abused in other ways by your partner or ex-partner” and, physical IPV, “You were kicked, hit, slapped or otherwise physically hurt by your partner or ex-partner.” Previous pregnancy cohort studies have utilised 2 similar items on physical and emotional IPV (Bowen et al., 2005; Flach et al. 2011).

To assess the incidence of stressful life events other than emotional and physical IPV, women who reported at least one of the remaining 22 specific stressful events at each timepoint were coded 1 (0 = *no stressful life events reported*).

#### Depression

The Structured Clinical Interview for the Diagnostic and Statistical Manual of Mental Disorders, Fourth Edition was administered at recruitment (i.e., early pregnancy) to assess for current depressive disorder (coded 1). Depressive disorders assessed by the SCID-IV include major depression, dysthymia and depression not otherwise specified. Depressive symptoms were measured at each timepoint using the Edinburgh Postnatal Depression Scale (EPDS; Cox et al. 1987). The EPDS comprises ten items measuring depressive symptoms and is valid for use with Australian women during the perinatal period (Boyce et al. 1993). In our sample, the EPDS scale at each measurement demonstrated strong internal consistency, with Cronbach’s alphas ranging from 0.85 to 0.86. We dichotomised the sample at each timepoint based on an EPDS cut-off score of ≥ 13 (coded 1), which has been used as a screener to denote the possibility of depression with high sensitivity and specificity (Boyce et al. 1993).

#### Childhood trauma

The Childhood Trauma Questionnaire (CTQ) (CTQ-SF; Bernstein et al. 2003) was administered to assess mothers’ self-reported experiences of childhood trauma. We use the total trauma score in this paper, where scores ≥ 37 indicate *moderate-to-severe childhood trauma* (coded 1) as indicated in the CTQ user’s manual (Galbally et al., 2019).

#### Covariates

Data were also collected at recruitment relating to maternal age (1 = *young maternal age, < 25 years*), tertiary education (1 = *university*), relationship status (1 = *married/de facto/stable partnership*), parity (1 = *nulliparous*), and ethnicity (1 = *European/Oceanic*).

### Statistical analyses

Descriptive statistics comparing those with clinical depression and not within the sample are presented examining if more women were young (under 25 years of age), partnered, were of Oceania ethnicity, and had completed tertiary education. For the first aim, to determine the prevalence of emotional and physical IPV during the perinatal period, we present the proportion of women who reported emotional IPV and, separately, physical IPV at each specific timepoint of data collection. We then display the prevalence of emotional and physical IPV at each timepoint by maternal childhood trauma and depression and assess the unadjusted associations using a series of cross-sectional *χ*^*2*^ tests of association. For the second aim, we test for changes in prevalence of IPV during the perinatal period, and if this change is associated with mental health, by fitting a series of nested Generalised Estimating Equation (GEE) models (binomial with log link) using a robust estimator of variance. Model coefficients (*b*) and adjusted risk ratios (*aRR*) with 95% confidence intervals are presented for each model. In these models, emotional and physical IPV were combined given the tiny prevalence of IPV – particularly physical IPV. In Model 1, the baseline trajectory of IPV risk over the four perinatal timepoints are estimated using time as a continuous variable. In Model 2, adjustments to this base trajectory of IPV risk are adjusted for young maternal age, tertiary education and parity. In Model 3, the maternal mental health measures of childhood trauma and depression are added with concurrent EPDS scores ≥ 13 and experiencing any other stressful life events. For the final model (i.e., Model 4), two-way interactions between time and both depressive disorder at recruitment and moderate-to-severe childhood trauma are included as predictors of IPV. However, non-significant interactions will be omitted from the final model presented. Significant interaction will be probed by plotting the model-estimated marginal probabilities and comparing within timepoint probabilities between groups. Significance is assessed using *p* < .05 and all statistics conducted using Stata 16 (StataCorp., 2019).

## Results

### Sample characteristics

The average age of women at recruitment was 31.73 (*SD* = 4.69) years ranging between 19 and 45 years. Table 1 displays sociodemographic characteristics of the sample and prevalence of mental health measures by depression. One-third of women (30.9%) reported moderate-to-severe trauma experienced in their childhood. Overall, the prevalence of specific childhood traumas was highest for moderate-to-severe emotional neglect (33.5%), followed by emotional abuse (29.3%), physical abuse (16.8%), physical neglect (14.1%) and lowest for moderate-to-severe childhood history of sexual abuse (12.1%). More than one-fifth (17.0%) of the sample met diagnostic criteria for a depressive disorder at recruitment, with most having met criteria for major depressive disorder (*n* = 80, 93.0%), 5 (5.8%) with dysthymia and 1 (1.2%) with depression not otherwise specified.

**Table 1:** Sample sociodemographic and mental health characteristics by depressive disorder (*N* = 505)

	No Depression *(n* = 419)	Depression (*n* = 86)	*p*-value
	n (%a)	n (%a)	
Young maternal age (< 25 years)	29 (6.9)	8 (9.3)	0.494^
Oceania/European	382 (91.2)	77 (98.5)	0.631^
Nulliparous	303 (72.3)	62 (72.1)	0.967
Married/de facto/stable partnership	388 (96.5)	83 (98.8)	0.486^
Tertiary education	**250 (59.7)**	**63 (73.7)**	**0.018**
Moderate-to-severe childhood trauma	**116 (27.7)**	**40 (46.5)**	**< 0.001**
*EPDS* ≥ *13*			
Early pregnancy	**26 (6.2)**	**29 (33.7)**	**< 0.001**
Third trimester	**21 (5.2)**	**27 (32.1)**	**< 0.001**
Six months postpartum	**32 (8.9)**	**13 (17.8)**	**0.023**
12 months postpartum	**27 (6.6)**	**21 (25.6)**	**< 0.001**
*Any stressful life events other than IPV*			
Early pregnancy	**234 (55.8)**	**66 (76.7)**	**< 0.001**
Third trimester	**208 (51.4)**	**62 (72.9)**	**< 0.001**
Six months postpartum	193 (53.3)	45 (59.2)	0.348
12 months postpartum	215 (52.3)	52 (62.7)	0.085

^a^ Valid % presented due to univariate missing data

^^^ Fisher’s exact test due to expected cell counts < 5; Bolded values have reached significance assessed using *p* <0.05

EPDS, Edinburgh Postnatal Depression Scale

### IPV and maternal mental health

**Fig. 1:**
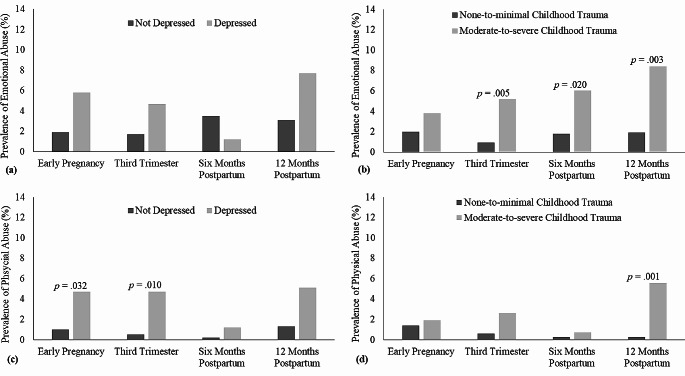
Prevalence of maternally reported emotional abuse at each timepoint by (**a**) depression and by (**b**) childhood trauma, and prevalence of maternally reported physical abuse at each timepoint by (**c**) depression and by (**d**) childhood trauma. Comparisons are made using Fisher’s exact χ2 tests of association

The prevalence of reported emotional IPV and physical IPV are displayed in Fig. 1. Figure 1(a and c) show that although reports of emotional IPV at each timepoint during the perinatal period, which included in early and late pregnancy and then at six and 12 months postpartum, did not differ significantly between women with and without a depressive disorder, depression was associated with significantly higher prevalence of physical IPV at early pregnancy (4.7% cf. 1.0%) and third trimester (4.7% cf. 0.5%). Despite the differences in physical IPV during pregnancy, there was no observable increasing trend in prevalence associated with depression. In contrast, moderate-to-severe childhood trauma compared to none-to-minimal childhood trauma was associated with significantly higher prevalence of emotional IPV at third trimester (5.2% cf. 0.9%), six months postpartum (6.0% cf. 1.8%) and 12 months postpartum (8.4% cf. 1.9%), and physical IPV at 12 months postpartum (5.6% cf. 0.3%). There was an observable increasing trend in the prevalence of IPV during the perinatal period for women who reported moderate-to-severe childhood trauma, which was more discernible for emotional IPV (3.8%, 5.2%, 6.0%, 8.4%) than physical IPV (1.9%, 2.6%, 0.7%, 5.6%).

### Predicting perinatal IPV

Table 2 presents the robust Generalising Estimating Equation (GEE) coefficients for the variables predicting IPV for each additive model. In Model 1, time was not a significant predictor of IPV, suggesting that the average risk of IPV did not change during the perinatal period from the model-estimated base risk of 3%. After adjusting for young maternal age (< 25 years), a tertiary education and parity in Model 2, and after the addition of mental health variables in Model 3, time remained a non-significant predictor of IPV. In Model 3, moderate-to-severe childhood trauma, any concurrent stressful life events other than IPV and concurrent EPDS scores greater than 12 were each independently associated with significantly higher risk of reporting IPV during the perinatal period (*p*’s < 0.05). For the final model, only the two-way interaction between time and moderate-to-severe childhood trauma was significant. Thus, the interaction between time and depressive disorder at recruitment was omitted. Model 4 estimates remained consistent with those from Models 1 to 3, such that being younger than 25, nulliparous, tertiary educated and having a depressive disorder at recruitment were each not significantly associated with the risk of reporting IPV during the perinatal period (*p*’s > 0.05). Further, concurrent EPDS scores greater than 12 and reporting other concurrent stressful life events were each independently associated with significantly higher probabilities of reporting IPV (both *p*’s < 0.001).

**Table 2:** Results of the robust generalised estimating equation model predicting IPV (*N* = 505)

	Model 1		Model 2		Model 3		Model 4	
	*b* *(robust se)*	*aRR* *(95%CI)*	*b* *(robust se)*	*aRR (95%CI)*	*b* *(robust se)*	*aRR* *(95%CI)*	*b* *(robust se)*	*aRR* *(95%CI)*
Constant (base risk)	-3.64 (0.31)	0.03 (0.01, 0.05)	-4.01 (0.56)	0.02 (0.01, 0.05)	-6.10 (0.65)	0.002 (0.006, 0.008)	-5.27 (0.67)	0.01 (0.001, 0.02)
Time	0.10 (0.10)	1.10 (0.91, 1.33)	0.09 (0.10)	1.09 (0.90, 1.32)	0.13 (0.09)	1.14 (0.96, 1.36)	− 0.17 (0.15)	0.84 (0.63, 1.13)
Young maternal age (< 25 years)			0.53 (0.59)	1.69 (0.53, 5.37)	0.27 (0.58)	1.31 (0.42, 4.10)	0.23 (0.59)	1.26 (0.40, 3.98)
Nulliparous			0.05 (0.40)	1.05 (0.48, 2.30)	− 0.25 (0.36)	0.78 (0.38, 1.57)	− 0.29 (0.36)	0.75 (0.37, 1.50)
Tertiary Education			0.46 (0.34)	1.58 (0.81, 3.12)	0.29 (0.36)	1.34 (0.66, 2.73)	0.27 (0.36)	1.31 (0.65, 2.67)
Moderate-to-severe childhood trauma					0.85** (0.32)	**2.34** **(1.24, 4.41)**	− 0.45 (0.60)	0.64 (0.20, 2.09)
Depressive disorder at recruitment					0.04 (0.37)	1.04 (0.50, 2.14)	0.05 (0.36)	1.05 (0.52, 2.12)
EPDS ≥ 13^					0.89*** (0.22)	**2.44** **(1.59, 3.75)**	0.97*** (0.22)	**2.64** **(1.72, 4.03)**
Any stressful life events other than IPV^					2.19*** (0.45)	**8.92** **(3.67, 21.65)**	2.13*** (0.43)	**8.43** **(3.63, 19.58)**
*Interaction Term*								
Timepoint*Moderate-to-severe childhood trauma							0.49** (0.19)	**1.64** **(1.12 2.39)**

^^^ Time-varying covariates

IPV, intimate partner violence; b, regression coefficient; se, standard error; aRR, adjusted risk ratio; CI, confidence interval; EPDS, Edinburgh Postnatal Depression Scale

**p < .05, ***p < .001

**Fig. 2:**
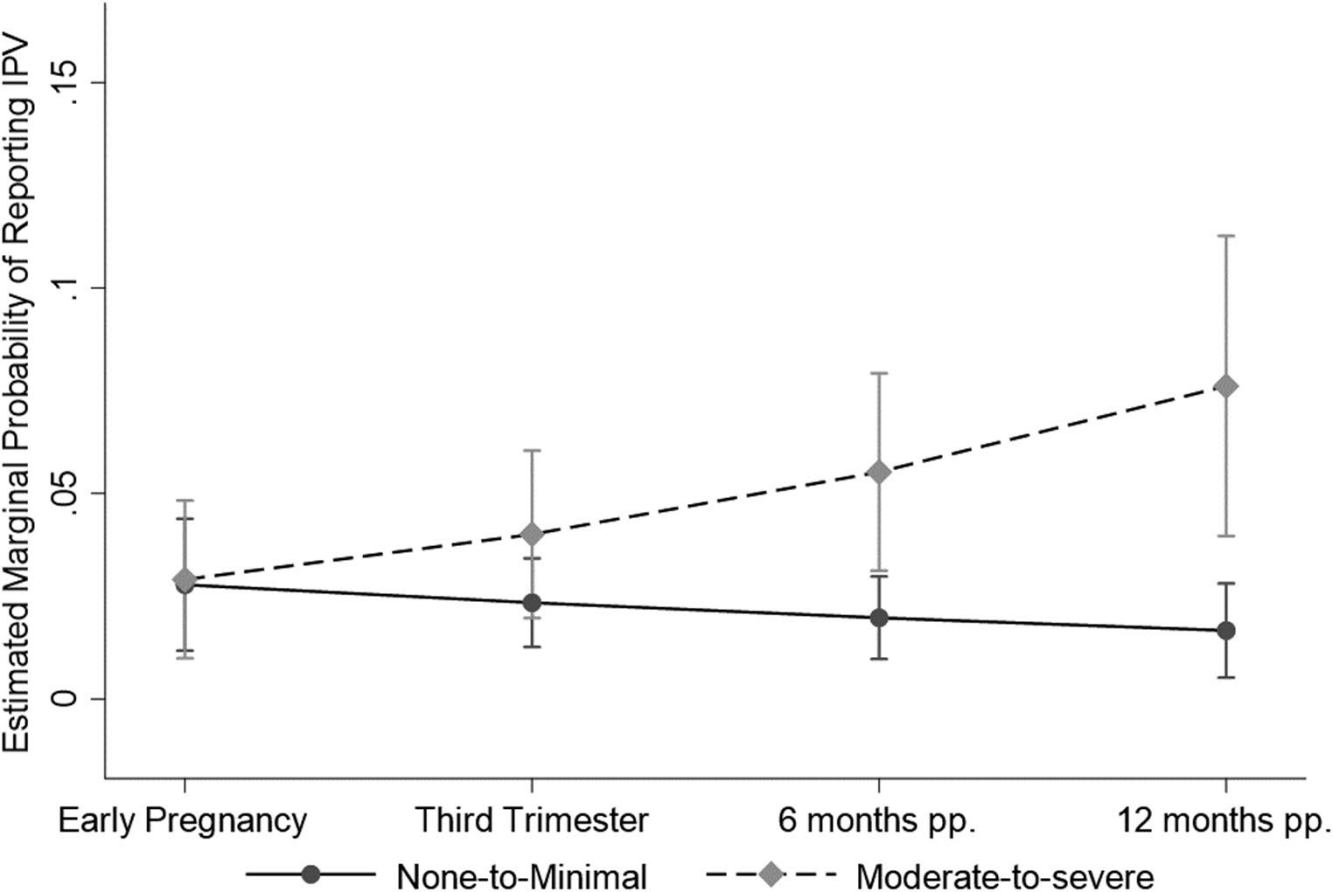
Marginal probabilities estimated by the robust GEE model predicting perinatal IPV for the none-to-minimal childhood trauma group and separately for the moderate-to-severe childhood trauma group. Error bars represent 95% confidence intervals

The interaction between time and maternal childhood trauma was significant (*p* = .011) in Model 4, such that mothers who reported moderate-to-severe childhood trauma had a significantly increasing adjusted risk of reporting IPV at each subsequent wave compared to women who reported none-to-minimal childhood trauma. This pattern is displayed in Fig. 2 using model-estimated marginal probabilities of IPV plotted for the childhood trauma groups at each timepoint. Relative to the none-to-minimal childhood trauma group at each timepoint, women who reported moderate-to-severe childhood trauma demonstrated no significant difference in the estimated probability of reporting IPV during pregnancy (early pregnancy: Δ = 0.001, *p* = .921; third trimester: Δ = 0.02, *p* = .158), but significantly higher estimated probability of reporting IPV at six months postpartum (Δ = 0.04, *p* = .007) and again at 12 months postpartum (Δ = 0.06, *p* = .002).

## Discussion

This study examined the prevalence of physical and emotional IPV in women with clinical depressive disorders in pregnancy compared to those without, and found there was no increase in emotional IPV in those with clinical depressive disorders, although there was a difference in higher reported physical IPV experienced in women with depressive disorders in early pregnancy. Furthermore, those experiencing IPV had higher depressive symptoms across the perinatal period as measured on the EPDS, as well as higher stressful events (other than IPV), and the association between IPV and depressive symptoms is consistent with previous studies that have predominantly examined depressive symptoms rather than disorder (Howard et al. 2013; Paulson 2022). This study also found that those with a history of moderate to severe childhood trauma were more likely to experience both emotional and physical IPV and this was more prominent in the postpartum.

While the diagnosis of a depressive disorder in early pregnancy was not associated with an increased risk of experiencing emotional IPV, physical IPV was associated with depression. In fact, the experience of any IPV was associated with increased depressive symptoms across pregnancy and the postpartum, placing those women at increased risk of developing depression and anxiety disorders over this period of early parenting. A systematic review examining perinatal depression and IPV previously found an association between IPV and depressive symptoms, and in addition this review found that different types of IPV were independently associated with depressive symptoms however this review noted there were few studies that had repeat measurement of depressive symptoms and IPV across the perinatal period, included a diagnostic measure with the majority utilising EPDS alone and few considered other variables associated with social adversity such as stressful life events and childhood trauma (Paulson 2022). Our findings included current other stressful life events and childhood trauma experiences and both were significantly associated with experiences of IPV. In studies outside of the perinatal period, child maltreatment history has been associated with later IPV in adulthood (Renner and Slack 2006; Shields et al. 2020). While we do not have the data to understand this relationship, it could be speculated that this may be due to the specific vulnerability of women experiencing both current psychosocial stress, as well as those who have experienced early developmental trauma. This vulnerability may be exacerbated at this time through an increase in reliance on partners financially, as well as for practical and emotional support, and being less easily able to leave home while pregnant or when caring for a young baby. It raises the question about whether early childhood trauma experiences as well as current stressful events and circumstances should be routinely asked within maternity care (e.g., the psychosocial screening measure the Antenatal Risk Questionnaire includes this within this measure), and furthermore, whether women might find it easier to disclose childhood trauma than current IPV (Austin et al. 2008). These findings reinforce the consistent call for trauma informed maternity and maternal child health care (Drexler et al. 2022). Given the importance of pregnancy and the postpartum as a time of social and psychological role transition, as well as the development of the early parenting relationship, understanding the unique and specific support needs for these women is critical (Austin et al. 2019; Drexler et al. 2022). .

Despite this study having women with depressive disorders and across the sample approximately 30% of women had a history of moderate to severe childhood trauma, there were relatively low rates of IPV reported, ranging from 1 to 8% across the perinatal period for physical and emotional abuse. In contrast, a community sample of Australian women across pregnancy and the postpartum, found 17% of women experienced IPV, but data on depression or childhood trauma was not reported (Gartland et al. 2011). Conversely, a study of routine enquiry of domestic violence by midwives, found 1.8% of women experienced domestic violence in early pregnancy, which rose to 5.8% in late pregnancy, reflecting our own study findings more closely (Bacchus et al., 2004). Similarly, a longitudinal study reported 1% of physical abuse in early pregnancy, rising to 2.9% in late pregnancy, however, those with five or more additional adversities were significantly more likely to experience IPV (Bowen et al., 2005). It is unclear whether the rates of IPV in our sample are reflective of the lower levels of social adversity in our sample, the narrower collection of only physical or emotional abuse (and not sexual violence or controlling or coercive behaviours) and/or reporting bias. It is important to note that physical IPV experiences were associated with depression, and any IPV was associated with an increase in depressive symptoms, thereby reflecting the impact of IPV on the mental health and wellbeing of these women. While some studies have found that those with clinical mental disorders such as depression may be more vulnerable to experiencing IPV, few studies utilise diagnostic measures of mental disorders. Moreover, beyond mental disorders, there will be other factors that are associated with an increase in a woman’s vulnerability to experiencing IPV in relationships. However, what is clear is the impact that IPV has on mental health, including depressive symptoms, as well as depression being associated with physical IPV.

A limitation of this study is the gap in data on controlling and coercive behaviours and experiences of sexual violence as forms of IPV. While IPV was measured using items previously established in studies, there are more comprehensive measures of IPV that cover a broader range of experiences of IPV, including, coercive control and sexual violence. An advantage is the repeat measurement of IPV experiences, though there remains a likelihood of underreporting of IPV when relying on self-report measures, including that used in this study.

Our study builds on this important area of research examining the relationship between IPV and perinatal depression and highlights the impact of IPV on mental health including increased symptoms of perinatal depression, as well as vulnerability to IPV associated with childhood trauma and current stressful life events. Finally, our study findings highlight the importance of trauma informed care for women in pregnancy and early parenting given the relationship between early childhood trauma, IPV, depression, and depressive symptoms, and the need to consider how to support the likely complex psychosocial needs of women who have experienced multiple forms of trauma at this critical time. Future research across the perinatal period is recommended to extend and clarify the findings in this study with a broader measure of IPV, as well as this study’s diagnostic and symptomatic measures of mental health and including women who have clinical mental disorders. This extension of our study would enable us to continue to build a better understanding of the associations and impacts of IPV and mental health in the perinatal period, information that is necessary to inform clinical practice for both maternity and perinatal mental health services.

## Electronic supplementary material

Below is the link to the electronic supplementary material.

Supplementary Material 1
