# Supplementary material for: Intimate partner violence across pregnancy and the postpartum and the relationship to depression and perinatal wellbeing: findings from a pregnancy cohort study
Source: Arch Womens Ment Health. 2024 Mar 9;27(5):807–15. doi: 10.1007/s00737-024-01455-z (PMC11405469; doi:10.1007/s00737-024-01455-z)
Supplement: Supplementary file 1 — Supplementary Material 1 [file 737_2024_1455_MOESM1_ESM.docx]

| **Reviewer Comment** | **Response** |
| --- | --- |
|  | Dear Editors and Reviewers  Thank you for reviewing our revised paper and taking the time to provide a fourth round of constructive comments to improve the manuscript. We address all comments below. |
| **Reviewer 7**  Thank you for the opportunity to review this reviewed manuscript. The changes you have made have improved it considerably.  Some minor errors noted are Abstract: Write EPDS after its full name first before writing just EPDS in the successive lines Page 6, line no. 115: Include CTQ after a full name Page 7, line no. 127: Extra opening bracket Page 10, line no. 206-207: Use past tense instead of future tense. Page 14, line no. 303: 'both' repeated | Thank you and the minor errors have all been corrected in the manuscript. |
| **Reviewer 8** |  |
| Still needs a lot of editing due to grammatical mistakes and awkward sentences. I attached some edits. This too a long time to read and edit, and really needs one of the study personnel to review and edit more | Thank you and we have reviewed and revised the manuscript throughout. |
